# Genetically Engineered Alginate Lyase-PEG Conjugates Exhibit Enhanced Catalytic Function and Reduced Immunoreactivity

**DOI:** 10.1371/journal.pone.0017042

**Published:** 2011-02-14

**Authors:** John W. Lamppa, Margaret E. Ackerman, Jennifer I. Lai, Thomas C. Scanlon, Karl E. Griswold

**Affiliations:** 1 Thayer School of Engineering, Dartmouth College, Hanover, New Hampshire, United States of America; 2 Department of Biological Engineering, Massachusetts Institute of Technology, Boston, Massachusetts, United States of America; 3 Department of Biological Sciences, Dartmouth College, Hanover, New Hampshire, United States of America; 4 Program in Molecular and Cellular Biology, Dartmouth College, Hanover, New Hampshire, United States of America; East Carolina University School of Medicine, United States of America

## Abstract

Alginate lyase enzymes represent prospective biotherapeutic agents for treating bacterial infections, particularly in the cystic fibrosis airway. To effectively deimmunize one therapeutic candidate while maintaining high level catalytic proficiency, a combined genetic engineering-PEGylation strategy was implemented. Rationally designed, site-specific PEGylation variants were constructed by orthogonal maleimide-thiol coupling chemistry. In contrast to random PEGylation of the enzyme by NHS-ester mediated chemistry, controlled mono-PEGylation of A1-III alginate lyase produced a conjugate that maintained wild type levels of activity towards a model substrate. Significantly, the PEGylated variant exhibited enhanced solution phase kinetics with bacterial alginate, the ultimate therapeutic target. The immunoreactivity of the PEGylated enzyme was compared to a wild type control using *in vitro* binding studies with both enzyme-specific antibodies, from immunized New Zealand white rabbits, and a single chain antibody library, derived from a human volunteer. In both cases, the PEGylated enzyme was found to be substantially less immunoreactive. Underscoring the enzyme's potential for practical utility, >90% of adherent, mucoid, *Pseudomonas aeruginosa* biofilms were removed from abiotic surfaces following a one hour treatment with the PEGylated variant, whereas the wild type enzyme removed only 75% of biofilms in parallel studies. In aggregate, these results demonstrate that site-specific mono-PEGylation of genetically engineered A1-III alginate lyase yielded an enzyme with enhanced performance relative to therapeutically relevant metrics.

## Introduction

The major contributor to mortality in cystic fibrosis (CF) patients is pulmonary infection by the Gram-negative bacterium *P. aeruginosa*. The majority (∼75%) of CF-associated *P. aeruginosa* isolates exhibit a mucoid phenotype characterized by overproduction of alginate, an exopolysaccharide component of the biofilm matrix [1]. Bacterial alginate is one of the most studied *P. aeruginosa* virulence factors [2], with confirmed roles in protection of bacteria from host immune defenses [3], [4], exacerbation of inflammatory tissue damage [5], and contribution to bacterial resistance towards conventional antibiotic therapies [6], [7], [8]. In addition, alginate has been shown to increase the viscosity of mucosal secretions contributing to respiratory tract obstructions [9]. Considering its important role in the pathology of *P. aeruginosa* infections of the CF lung, alginate represents an attractive target for developing novel therapeutic agents for CF patients.

Alginate lyase enzymes (EC 4.2.2.3) efficiently degrade alginate *via* β–elimination cleavage of glycosidic bonds in the polymer backbone. Numerous observations support the hypothesis that alginate lyases could be powerful therapeutic agents for treating mucoid *P. aeruginosa* infections. For example, alginate lyases have been shown to enhance phagocytosis of *P. aeruginosa* by human macrophages [10], increase the susceptibility of *P. aeruginosa* to a variety of antibiotic treatments [6], [11], [12],[13], and decrease the viscosity of CF sputum [14]. The latter activity suggests a therapeutic application analogous to that of recombinant human DNase (Pulmozyme®), an inhaled enzyme therapy that degrades extracellular DNA, aids in clearance of viscous airway obstructions, and temporarily improves pulmonary function in CF patients [15]. Unfortunately, alginate lyases are invariably derived from non-human sources, and their exogenous origins may predispose them towards excessive immunogenicity in human patients. An immune response against biotherapeutic agents can manifest a spectrum of complications including increased rates of drug clearance, direct inhibition of therapeutic activity, and varying degrees of allergic reaction with the potential for life-threatening anaphylactic shock [16]. There is an increasing awareness of the risks associated with immune responses against biotherapeutic agents [17], and this knowledge is prompting the restructuring of biotherapeutic development strategies so as to address potential safety concerns earlier in the process [18]. Considering the tremendous potential of alginate lyase therapeutic agents, strategies to mitigate putative anti-enzyme immune reactions merit examination.

Chemical modification of therapeutic proteins with polyethylene glycol (PEG) is a common approach for modulating immunogenicity and stability [19]. Indeed, PEGylation of *Sphingomonas* sp. A1-III alginate lyase (A1-III), one therapeutic candidate, has been shown to reduce antibody binding *in vitro* [20]. Unfortunately, the random attachment of amine-reactive PEG molecules to solvent exposed lysines of A1-III resulted in a significant proportion of inactivated enzyme (>50% inactivation with 10 of 12 formulations). Thus, while PEGylation can successfully reduce the enzyme's immunoreactivity, maintaining a homogenous enzyme composition with high catalytic activity necessitates a more controlled PEG-conjugation strategy.

To facilitate precise control over both the site of PEG attachment and the extent of PEGylation, cysteine residues were engineered into the A1-III enzyme at five different surface accessible locations. These rationally substituted cysteine residues provided an orthogonal chemical handle for site-specific PEGylation reactions using maleimide activated PEG. It was anticipated that selective and controlled PEGylation would result in modified variants simultaneously demonstrating high catalytic proficiency and reduced immunoreactivity. In this study, solution phase reaction kinetics, biofilm disrupting activity, and *in vitro* antibody binding of genetically engineered PEG variants have been assessed and compared to the non-PEGylated wild type enzyme control. The results suggest that at least one modified enzyme meets or exceeds the experimental objectives, and thereby possesses enhanced potential as an antibacterial therapy.

## Results

### Construction of A1-III Mutants

**Figure 1:**
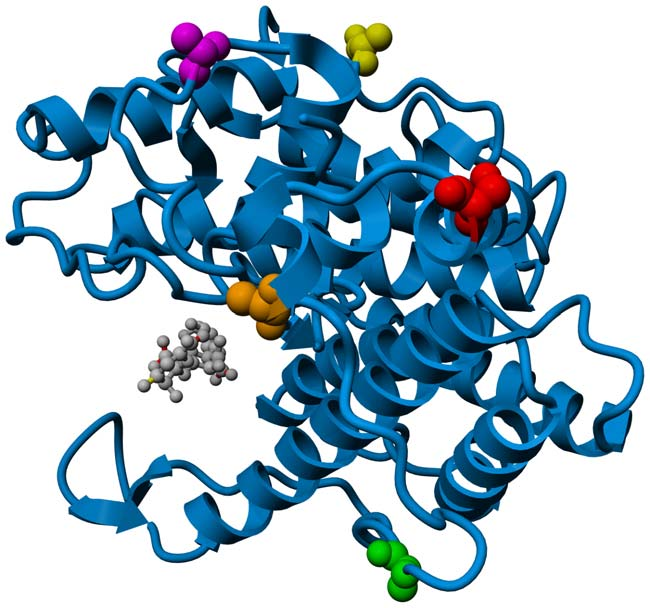
Sites of cysteine substitution. Ribbon diagram of alginate Lyase A1-III (PDB file 1HV6). A trisaccharide reaction product is bound in the active cleft and shown as a grey ball and stick model. Amino acid residues targeted for cysteine substitution are shown in space filling mode, and are color coded as follows: S32C = Red, A41C = Orange, A53C = Green, A270C = Yellow, and A328C = Purple.

To facilitate site-specific PEGylation, mutant A1-III genes encoding single cysteine substitutions were constructed by total gene synthesis [21]. The synthetic genes were codon optimized for expression in *E. coli*, and each gene encoded a single site-specific cysteine substitution: S32C, A41C, A53C, A270C or A328C, where residue numbering is per Yoon *et al*. [22] The five sites for mutation were selected based on an analysis of PDB structure 1HV6 [22]. Priority was placed on small amino acids that, when substituted with a cysteine, would result in a solvent exposed thiol group (Fig. 1 and Movie S1). Particular emphasis was given to residues with spatial proximity to the S32-C49 peptide segment, a motif that has previously been reported as constituting an immunodominant region of the enzyme [23]. A *C*-terminal hexahistidine tag (his-tag) was appended to each mutant to facilitate purification by immobilized metal-ion affinity chromatography (IMAC). A construct encoding the corresponding his-tagged version of the wild type enzyme (WT-his) was generated as a control.

Expression levels of the recombinant enzymes from a T7 driven pET vector system varied moderately. Following overnight shake flask induction, cell lysis, IMAC purification and dialysis, the WT-his enzyme and high yielding variants such as A53C-his produced upwards of 20 mg per liter of cell culture. In contrast, variant A41C-his yielded 3-fold less protein under the same expression conditions. Importantly, non-reducing SDS-PAGE gels showed that the cysteine variants were isolated predominantly as monomers. Only after extended storage were the genetically engineered proteins found to dimerize *via* intermolecular disulfide bond formation. Interestingly, the non-reducing SDS-PAGE analysis also indicated that one or both of the protein's two native disulfide bonds (C49–C112 and/or C188–C189) were not fully formed upon cell lysis and IMAC separation, but that oxidation to the fully disulfide bonded state occurred during the first 24 to 48 hours after purification (data not shown). This observation is consistent with expression of the enzymes in the reducing environment of the *E. coli* cytoplasm and subsequent oxidation by molecular oxygen following lysis and storage.

### PEG Conjugation and Purification

**Figure 2:**
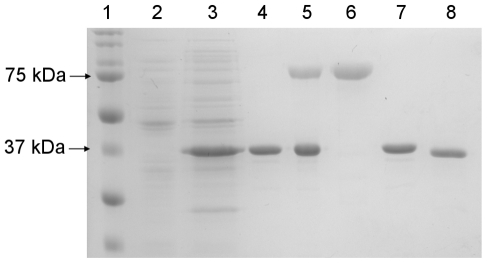
SDS-PAGE analysis of PEGylated variant production. Samples run on a reducing 12.5% gel, and stained for total protein with Coomassie brilliant blue. Lane 1: Bio-Rad Precision Plus Protein Ladder; Lane 2: Whole cell lysate of non-expressing cells; Lane 3: Whole cell lysate of induced cells; Lane 4: IMAC purified A53C-his; Lane 5: Crude A53C-his PEGylation reaction product; Lane 6: Size exclusion FPLC purified A53C-his-PEG; Lane 7: IMAC purified WT-his; Lane 8: FPLC purified native WT.

Exposed thiol groups of the engineered cysteine residues were conjugated to a 20 kDa methoxy-maleimide PEG. Reaction time, temperature and stoichiometry were the subject of detailed optimization studies, and it was ultimately determined that 1 hour reactions at 25°C with a 5∶1 molar ratio of PEG:enzyme typically yielded maximal mono-PEGylated product, i.e., protein molecules each bearing a single PEG polymer chain. Mono-PEGylated reaction products were readily separated from unconjugated protein by FPLC size exclusion chromatography (Fig. 2), and optimized reactions typically produced 40 to 50% yields of >95% pure material.

### Enzyme Kinetics

Alginate depolymerization kinetics were assessed for the various PEGylated enzymes and the WT-his control using brown seaweed alginate (BSWA) as a model substrate. Michaelis constants (K_m_) and maximum reaction velocities (V_max_) were determined by nonlinear regression of initial velocities vs. substrate concentration. All five of the PEGylated variants were found to possess catalytic efficiencies (V_max_/K_m_) exceeding that of the corresponding WT-his construct, although most exhibited a decrease of 2-fold or less in V_max_ (Table 1). Variant A41C-his-PEG was found to possess particularly low maximum reaction velocities, and it was therefore eliminated from further studies.

**Table 1:** Kinetic parameters for alginate degradation.

Enzyme	V_max_[ΔA_235_ (min · mg)^−1^]	K_m_(µg/ml)	V_max_/K_m_
WT-his	440±30	80±20	6±1
A53C-his	280±30	30±6	9±2
S32C-his-PEG	330±20	26±6	13±3
A41C-his-PEG	134±3	7.1±0.8	18±2
A53C-his-PEG	460±50	40±10	13±4
A270C-his-PEG	300±20	15±5	20±7
A328C-his-PEG	180±10	13±4	14±4

Depolymerization of BSWA was followed by monitoring absorbance at 235 nm. Kinetic parameters were determined by nonlinear regression of initial rate vs. substrate concentration data (Prism version 4.0).

One modified enzyme, A53C-his-PEG, maintained V_max_ values similar to the WT-his control, and the activity of this variant was examined in greater detail. To separate the effects of the point mutation from the effects of PEGylation, the kinetics of A53C-his were measured both before and after PEG conjugation (Table 1). The A53C amino acid substitution drove a reduction in both V_max_ and K_m_, but subsequent PEGylation produced a 60% increase in V_max_ restoring the variant's maximum reaction velocity to wild type levels while not altering the reduced K_m_ value. The result was an enzyme-PEG conjugate with a >2-fold improved catalytic efficiency compared to WT-his.

**Figure 3:**
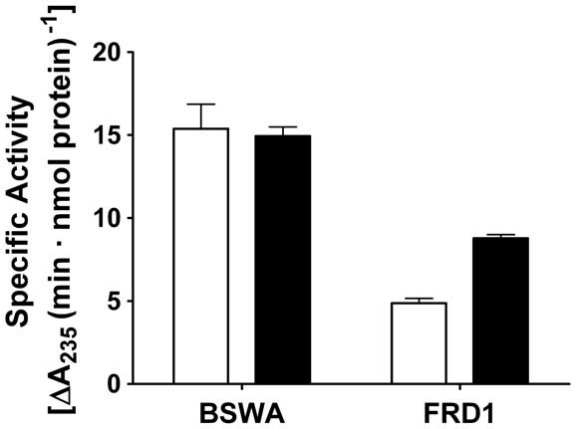
Comparison of reaction kinetics with BSWA and bacterial alginate. The specific activities of WT-his (white bars) and A53C-his-PEG (black bars) were determined with a model alginate substrate (BSWA) as well as with purified bacterial alginate (FRD1). The two enzymes are equally active with BSWA at saturating concentrations, but the PEGylated variant exhibits 80% faster kinetics with the bacterial substrate (p<0.01), which is the ultimate therapeutic target. Error bars represent standard deviation.

Alginate biopolymer produced by mucoid *P. aeruginosa* pathogens differs from that produced by brown seaweed in that bacterial alginate is partially acetylated at the C2 and C3 hydroxyls of mannuronate residues [24]. To evaluate activity on the bacterial substrate, alginate was purified from the mucoid *P. aeruginosa* clinical isolate FRD1. The specific activities of WT-his and A53C-his-PEG were determined using 0.1% (wt/vol) bacterial alginate. Unexpectedly, the PEGylated variant exhibited a 1.8-fold increased specific activity relative to the corresponding WT-his construct (Fig. 3).

It is possible that the altered catalytic activities of the PEGylated variants resulted from subtle structural perturbations to the enzyme's 3-dimensional fold. Such deviations from the native structure might have undesired consequences that compromise the potential for practical utility, e.g., decreasing enzyme stability during long term storage. To assess the impact of PEGylation on storage stability, the activity of A53C-his-PEG was followed during more than two months of storage at 4°C. No loss of activity was observed during the course of the 70 day experiment (data not shown).

### Antibody Binding and Immunogenicity

**Figure 4:**
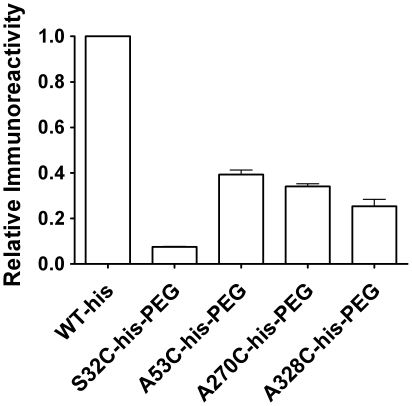
Immunoreactivity by ELISA. The antibody concentration required to achieve 50% maximum ELISA signal (EC_50_) was determined for each enzyme using polyclonal anti-A1-III antibody purified from rabbit immune serum. The results are reported as fractional immunoreactivity based on normalization with the WT-his enzyme, which was included as an internal control in all experiments (see Experimental Procedures). All of the PEGylated enzymes were found to exhibit significantly reduced antibody binding relative to the WT-his control (p<0.01 for each). Error bars represent standard deviation.

Polyclonal anti-A1-III IgG was purified by antigen affinity chromatography of pooled serum from two New Zealand white rabbits, which had both been immunized with the non-tagged, native enzyme (WT). The EC_50_ of the polyclonal antibody was determined for various PEGylated enzymes using standard ELISA techniques, and these values were compared to that for the non-PEGylated WT-his control. Genetically engineered variants S32C-his-PEG, A53C-his-PEG, A270C-his-PEG and A328C-his-PEG exhibited a 40–90% decrease in immunoreactivity relative to the WT-his enzyme counterpart (Fig. 4). Together, the high catalytic activity and decreased antibody binding of A53C-his-PEG distinguished this enzyme as a particularly promising candidate.

**Figure 5:**
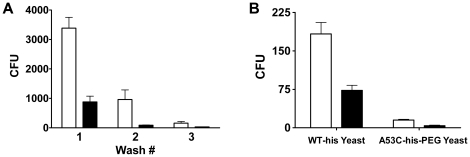
Human antibody binding. The WT-his (white bars) and A53C-his-PEG (black bars) protein targets were biotinylated and captured on the surface of streptavidin coated magnetic beads. A) The two bead preparations were independently incubated with a yeast surface displayed scFv antibody library derived from human immune cells. Following binding, the beads were magnetically separated and washed three times. The number of yeast that remained bound after each wash step was determined by plating serial dilutions of the resuspended beads and enumerating cfu's. The resulting yeast colonies represent human scFvs that specifically bound to the A1-III enzymes on the corresponding magnetic beads. A53C-his-PEG coated magnetic beads bound up to 13-fold fewer human antibodies than did the WT-his coated beads (p<0.01 for each of the three washes). B) Characterization of first round binders from both protein targets. Yeast isolated as binders to either WT-his or A53C-his-PEG were propagated and subsequently incubated with magnetic beads bearing each protein target. For both yeast populations, the A53C-his-PEG coated beads (black bars) bound at least 60% fewer cells than did the WT-his beads (white bars), a result that demonstrates PEGylation effectively blocked key immunogenic epitopes (p<0.01 for all differences). Error bars represent standard deviation.

To evaluate immunogenicity by a metric with greater relevance to human patients, binding of a naïve human antibody repertoire to both WT-his and A53C-his-PEG was assessed. Each protein was biotinylated and immobilized at saturating mM surface densities on streptavidin-coated magnetic beads. These alginate lyase coated beads represent one of two key elements in the immunogenicity assays. The second element is a yeast library displaying 10^9^ human scFv antibody fragments [25]. Yeast surface display produces a high degree of scFv multivalency, and when mixed with the alginate lyase coated beads, the resulting avid interactions facilitate capture of low affinity binders likely present in the human immune repertoire prior to affinity maturation. As a result, yeast cells expressing an scFv that recognizes an epitope on the candidate proteins are bound to the surface of the magnetic beads. Following a pre-screen to remove non-specific binders, the yeast library was incubated separately with either WT-his coated beads or A53C-his-PEG coated beads. After binding of the library, the beads were magnetically separated, unbound yeast in the supernatant were removed by aspiration, and the beads were resuspended in fresh buffer. An aliquot of this bead slurry was serial diluted, plated on yeast growth media, and outgrown to determine the number of colony forming units (cfu's) that remained bound to the beads. The washing and plating procedure was repeated two additional times, and the number of bead-bound yeast was determined for each wash step. The resulting cfu counts provide a means to assess the relative reactivity of a human antibody repertoire towards the two target proteins. Note that each yeast colony represents a single human scFv antibody fragment that specifically bound the target protein on the cognate magnetic bead surface. Beads coated with the PEGylated enzyme target were found to bind up to 13-fold fewer yeast cells than those coated with the WT-his enzyme (Fig. 5a, wash 2). Because the A53C-his and WT-his proteins are nearly identical in amino acid sequence, the difference in binding counts indicates that the PEG moiety effectively blocks interactions between human scFvs and their corresponding immunogenic epitopes on the A1-III enzyme.

Bound yeast cells isolated during these initial experiments represent enriched populations displaying scFvs that specifically recognize epitopes of either WT-his or A53C-his-PEG. To assess the cross-reactivity of the scFvs, yeast selected as binders to the WT-his beads were propagated and employed in a second round of binding experiments against both proteins in parallel. Likewise, yeast that initially bound the A53C-his-PEG beads were similarly tested for cross-reactivity. Importantly, yeast originally isolated as binders to the WT-his enzyme had a reduced capacity to recognize the PEGylated variant. Furthermore, yeast originally isolated as binders to A53C-his-PEG more readily recognized the WT-his protein then their original PEGylated target (Fig. 5b). Collectively, this data set implies that, although the A1-III enzyme's human antibody epitopes have not been completely occluded, PEGylation effectively reduces access to these sites. In particular, site specific PEGylation of A1-III alginate lyase (i) substantially reduced binding of naïve human antibody repertoires (Fig. 5a), and (ii) blocked >50% of specific, human, anti-A1-III scFv antibody fragments (Fig. 5b).

### Biofilm Disruption Studies

**Figure 6:**
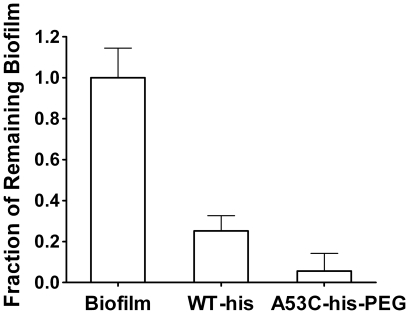
Disruption of mucoid *P. aeruginosa* biofilms. Adherent biofilms of a mucoid clinical isolate were established in 96-well plates and subsequently treated with 1 mg/ml enzyme for 1 hour. Remaining biofilm was then quantified using an alginate-sensitive lectin-HRP conjugate and ABTS substrate. Signals were normalized to a buffer only treatment. Both enzymes removed a significant proportion of biofilm relative to the buffer control (p<0.01). Importantly, theA53C-his-PEG enzyme removed >15% more biofilm than the WT-his enzyme (p<0.025). Error bars represent standard deviation.

There exists considerable evidence that *P. aeruginosa* grows in biofilm communities during CF lung infection [26], and it is likely that disrupting alginate biofilms represents a key challenge in the fight to eradicate CF-associated *P. aeruginosa* infections. To assess this therapeutically relevant aspect of enzyme function, biofilms of the alginate-producing *P. aeruginosa* strain Xen5, a derivative of clinical isolate ATCC 19660, were first established by growth in 96-well plates. Subsequently, adherent biofilms were treated for one hour with 1 mg/ml of WT-his or A53C-his-PEG and then washed to remove degraded biofilm. The remaining adherent alginate biofilm matrix was quantified using a ConA lectin-HRP conjugate that binds to mannuronate residues of alginate [27]. The percentage of biofilm removed by each enzyme was determined by comparison to wells receiving a buffer control treatment. Both the wild type and PEGylated enzymes were found to effectively remove the majority of established biofilm from the wells (Fig. 6). Consistent with its enhanced solution phase activity towards bacterial alginate, A53C-his-PEG exhibited a significant (p = 0.025) increase in mucoid biofilm disruption relative to the WT-his protein (94% vs. 75% biofilm removal, respectively). These results suggest that the enhanced catalytic performance of the genetically engineered A53C-his-PEG enzyme may have relevance to clinical applications.

## Discussion

Biofilms are thought to play a key role in refractory *P. aeruginosa* infections of the CF airway [26]. In particular, the transition of *P. aeruginosa* to a mucoid phenotype is associated with alginate overproduction, altered biofilm architecture, high level antibiotic-resistance, and accelerated deterioration of lung function [1], [8]. As a consequence, inhaled alginate lyase enzymes could represent powerful new therapies for treating CF lung infections. To realize their therapeutic benefit in humans, however, the risks associated with the putative immunogenicity of these heterologous enzymes should be appropriately mitigated. It has been shown previously that random PEGylation of free amines on the surface of A1-III alginate lyase effectively blocked enzyme-specific antibody binding [20]. Unfortunately, this strategy resulted in a significant loss of catalytic proficiency, as most preparations exhibited >50% reduction in alginate degrading activity. For a given PEG chain length, the degree of inactivation was directly proportional to the degree of de-immunization, a result that likely derived from the stochastic nature of NHS-ester conjugation to protein surfaces.

To better leverage the de-immunizing properties of PEG, a site-specific PEGylation strategy has been developed for the A1-III enzyme. Several surface accessible residues of the native enzyme were substituted with cysteines, and site-specific mono-PEGylation of the genetically engineered variants was achieved using a maleimide-activated 20 kDa PEG chain. Importantly, all of the site-specific, mono-PEGylated variants examined here were found to possess improved catalytic efficiency with a model substrate, BSWA. Maximum catalytic activity, however, was found to be critically dependent on the exact site of PEG attachment, as only variant A53C-his-PEG was found to maintain a V_max_ comparable to that of the wild type enzyme control. This outcome underscores a fundamental advantage of orthogonal conjugation chemistry: the site and extent of protein modification can be precisely controlled so as to yield a homogeneous enzyme preparation having uniformly high functionality.

Of particular relevance to treatment of bacterial infections, A53C-his-PEG was 80% more active than the WT-his control when assayed against solutions of bacterial alginate. The mechanistic origins of this enhanced activity on bacterial but not BSWA are not entirely clear. It is possible that PEGylation simply distorts the native enzyme structure so as to better accommodate the bulkier, acetylated, bacterial alginate. An alternative explanation, however, could relate to the acetylated alginate's greater hydrophobicity and increased extent of intermolecular interaction [28]. A high degree of substrate-substrate interaction could reduce enzyme accessibility to individual alginate chains and slow substrate degradation relative to non-acetylated BSWA. The amphipathic nature of PEG allows it to interact closely with both hydrophilic and hydrophobic molecules [19], and this property could facilitate insertion into amorphous higher-order structures of acetylated bacterial alginate. We speculate that the PEG moiety of A53C-his-PEG may disrupt enhanced substrate-substrate interactions in the enzyme's local environment, and thereby free individual alginate chains for more efficient enzymatic degradation. Loose parallels might be drawn to cellulases and chitinases, which efficiently degrade highly ordered, macromolecular, carbohydrate substrates. To do so, these enzymes employ non-catalytic binding domains that disrupt intermolecular polymer packing and enhance access to individual substrate chains [29], [30]. Certainly, this analogy should be approached with caution, as alginate solutions are hydrogels as opposed to crystalline or semi-crystalline substrates. None-the-less, site-specific PEGylation of A1-III alginate lyase has yielded a functionally enhanced enzyme that degrades bacterial alginate with greater efficiency.

In addition to maintaining high level catalytic activity, de-immunization of the A1-III protein was a second critical design objective of these experiments. The PEGylated constructs showed a 60–90% reduction in immunoreactivity with rabbit anti-A1-III IgG antibodies. Of greater relevance to human use, A53C-his-PEG bound a substantially smaller fraction of a naïve human scFv antibody library, relative to the non-PEGylated WT-his control. Furthermore, human scFvs that specifically bound the WT-his enzyme were 2.5-fold less likely to bind A53C-his-PEG. These data suggest that site-specific PEGylation has yielded a general reduction in the enzyme's antibody reactivity, and the studies with human antibody fragments lead us to hypothesize that the reduced immunoreactivity could translate to the clinic.

In clinical applications of alginate lyases, high level solution-phase activity may not be sufficient to affect a therapeutic benefit in CF patients. Instead, the practical utility of alginate lyase therapies will likely be defined by their capacity to disrupt mucoid *P. aeruginosa* biofilms. During a one hour treatment, the modified A53C-his-PEG enzyme removed more than 90% of established biofilms, a >15% improvement over the non-PEGylated WT-his protein. This enhanced ability to disrupt biofilms is consistent with the improved solution-phase kinetics of the engineered enzyme, and may stem from a similar mechanistic origin. While biofilms in the human airway have properties distinct from those grown on abiotic surfaces [26], the fact that A53C-his-PEG virtually cleared adherent mucoid biofilms suggests that it or similar enzymes could yield therapeutic benefits in the treatment of CF associated, *P. aeruginosa* infections.

## Materials and Methods

Oligonucleotides were purchased from IDT (Coralville, IA), and were purified by standard desalting. The gene sequences for the A1-III alginate lyase enzymes were derived from the wild type A1-III enzyme encoded in the genome of *Sphingomonas* sp. A1 (GenBank: AB011415). Restriction enzymes, Phusion polymerase, and T4 ligase were from New England Biolabs (Ipswich, MA), and were used as directed by the manufacturer. Expression vector pET28b was from Novagen (San Diego, CA). Plasmid purification kits, Ni-NTA agarose and corresponding columns were from QIAGEN (Valencia, CA). Gel extraction/DNA clean up kits were from Zymo Research (Orange, CA). 20 kDa methoxy-maleimide polyethylene glycol (PEG) was from JenKem Technology (Allen, TX). ÄKTA FPLC system and Superdex75 SEC resin were from GE Healthcare Life Sciences (Piscataway, NJ). Concanavalin A-horseradish peroxidase conjugate (ConA-HRP), medium viscosity brown seaweed alginate (BSWA) (cat #A2033), and 2,2′-azino-bis(3-ethylbenzthiazoline-6-sulphonic acid) (ABTS) were from Sigma-Aldrich (St. Louis, MO). BCA assay and AminoLink Plus Immobilization Kits were from Pierce Biotechnology (Rockford, IL). Polyclonal goat anti-rabbit HRP conjugate antibody was from Millipore (Billerica, MA). All other reagents were from Fisher Scientific (Pittsburgh, PA), unless specifically noted.

### Data Analysis

Experiments were conducted in triplicate unless otherwise noted, and statistical significance was determined using two-tailed t-tests.

### Ethics Statement

This study was carried out in strict accordance with the recommendations in the Guide for the Care and Use of Laboratory Animals of the National Institutes of Health. The protocol was approved by the Institutional Animal Care & Use Committee of Dartmouth College (Protocol Number: 07-07-11CL), and all efforts were made to minimize suffering.

### Construction and Cloning of A1-III Encoding Genes

Following the procedure of Hoover and Lubkowski [21], synthetic genes, codon optimized for expression in *E. coli*, were assembled for both the wild type [31] and cysteine point mutant A1-III enzymes. The genes were appended with a 5′-methionine codon, a 5′-FatI restriction site spanning the ATG start, and a 3′-XhoI restriction site immediately following the terminal serine codon (appends a non-native, *C*-terminal LeuGlu sequence). Each point mutant gene encoded a single cysteine substitution at serine 32, alanine 41, alanine 53, alanine 270, or alanine 328 (numbering as per Yoon *et al*. [22]). The 1,089 base pair synthetic A1-III genes were digested with FatI and XhoI, and ligated into NcoI and XhoI digested pET-28b expression vector resulting in an in frame fusion with the hexahistidine tag encoded by the plasmid. Ligations were transformed into electrocompetent DH5alpha [F^−^Φ80*lac*ZΔM15Δ(*lac*ZYA*-arg*F)U169*rec*A1*end*A1*hsd*R17(r_K_^−^m_K_^+^) *pho*A*sup*E44 *thi*-1*gyr*A96*rel*A1 λ^−^], and the identities of the cloned genes were verified by sequencing plasmid isolated from individual clones. These plasmid constructs encoded his-tagged wild type (WT-his), or point mutant A1-III enzymes (S32C-his, A41C-his, A53C-his, A270C-his, and A328C-his). A gene encoding an untagged version of the wild type (WT) enzyme was constructed in a similar manner, but insertion of a dual stop codon (TGATAG) before the 3′ restriction site terminated translation prior to the hexahistidine coding sequence. Sequence verified plasmids were subsequently transformed into electrocompetent HMS174(DE3) expression hosts [F^−^ *recA1hsdR*(r_K12_^−^ m_K12_^+^) (DE3) (Rif^R^)]. The expression host also bore the pLysS plasmid to repress basal expression.

### Protein Expression and Purification

Overnight cultures of expression hosts were grown in LB supplemented with30 µg/ml kanamycin and 34 µg/ml chloramphenicol at 37°C, and then sub-cultured 1∶100 into 500 ml of fresh media. Cultures were grown at 37°C to mid-log, equilibrated to 25°C, and induced with 0.5 mM IPTG for 20 hours. Following induction, cell cultures were centrifuged at 5000*g*, 4°C for 10 minutes, pellets were resuspended in 5 ml of native lysis buffer (50 mM NaH_2_PO_4_, 300 mM NaCl, 10 mM Imidazole, pH 8.0), transferred to a 10 ml Pyrex beaker, and equilibrated on ice for 20 minutes. Cells were disrupted by sonication (Fisher 550 Sonic Dismembrator). Whole cell lysate was dispensed into 2 ml eppendorf tubes and centrifuged at 17,000*g*, 4°C, for 20 minutes. The supernatant was removed, syringe filtered through a 0.22 µm PES membrane, and gently mixed with a 0.4 ml bed volume of Ni-NTA agarose, which had been equilibrated with native lysis buffer. After binding at 4°C for 1 hour, the column was drained and washed with 10 bed volumes of wash buffer (50 mM NaH_2_PO_4_, 300 mM NaCl, and 20 mM imidazole pH 8.0). Purified A1-III was eluted in a native elution buffer (50 mM NaH_2_PO_4_, 300 mM NaCl, 250 mM imidazole, pH 8.0), dialyzed into storage buffer (20mM NaH_2_PO_4_ pH 6.5), and kept at 4°C. The purity of enzyme preparations was typically >95% as assessed by Coomassie-stained SDS-PAGE gels. Enzyme concentrations were routinely determined by A_280_ (NanoDrop 1000, Thermo Scientific, Waltham, Ma) using a standard curve that had been independently validated by BCA assay.

For the purpose of immunizing rabbits for antibody production, the non-tagged native enzyme (WT) was purified as described previously [32]. Enzyme purity was >99% as assessed by Coomassie-stained SDS-PAGE gels, and enzyme solutions were stored in phosphate buffered saline (PBS) at 4°C prior to use.

### Covalent Conjugation to PEG

Preliminary optimization studies with the A53C-his point mutant examined the effects of time (5 minutes to overnight), temperature (25°C to 37°C), and stoichiometry (1∶1 to 20∶1 PEG:protein molar ratio) as reaction variables, and it was ultimately determined that 1 hour reactions at 25°C with a 5∶1 molar ratio of PEG:enzyme typically yielded maximal mono-PEGylated product, i.e. protein molecules each bearing a single PEG polymer chain. Subsequently, purified A1-III cysteine mutants were covalently coupled with a 5 molar excess of 20 kDa methoxy-maleimide PEG. PEG was initially solubilized in DMSO at a concentration of 100 mg/ml, and 12 µl were added to 500 µl of a 1 mg/ml enzyme solution in 20 mM NaH_2_PO_4_ pH 6.5. Reactions were incubated at room temperature for 1 hour, and then loaded onto a 120 ml bed volume Superdex 75 size exclusion column. The column was eluted with 150 mM NaCl, 50 mM NaH_2_PO_4_ pH 7.0 at a flow rate of 0.6 ml/min. Mono-PEGylated A1-III product eluted at ∼53 ml. Enzyme purity was typically >95% as assessed by Coomassie-stained SDS-PAGE gels, and enzyme solutions were stored at 4°C for later use. The concentrations of PEGylated enzymes were determined by A_280_, as independent experiments demonstrated that conjugation to the PEG moiety did not alter enzyme molar absorptivity.

### Enzyme Kinetic Analysis

Enzymatic activities were assessed in a 96-well plate format. Briefly, 5 µl of purified enzyme was added to each of 12 contiguous wells in a UV transparent, 96-well plate (Costar, Fisher #3635). Using a 12-channel pipette, 195 µl of alginate in reaction buffer (150 mM NaCl, 50 mM NaH_2_PO_4_ pH 7.0) was simultaneously added to each of the wells. BSWA concentrations were varied from 0.001% to 0.05% (wt/vol), and each concentration was assayed in triplicate. The 96-well plates were immediately transferred to a UV/Vis plate reader (SpectraMax 190, Molecular Devices, Sunnyvale, CA), and product formation was monitored by measuring absorbance at 235 nm every 15 seconds for 10 minutes. Initial velocities were taken from the linear portions of the absorbance verses time curves, and V_max_ and K_m_ values were determined by non-linear regression of initial reaction rates verses substrate concentration. Specific enzyme activities towards bacterial alginate, purified from *P. aeruginosa* FRD1 as described previously [33], were determined in triplicate at 0.1% (wt/vol) substrate concentration. Assays were carried out essentially as described above.

### IgG Antibody Immunoreactivity

A1-III alginate lyase antiserum was obtained from Covance Research (Denver, PA). Two New Zealand white rabbits were initially immunized by subcutaneous injection of 250 µg of purified WT A1-III mixed with Freund's complete adjuvant (FCA). At twenty day intervals, the rabbits were boosted with subcutaneous injections of 125 µg of purified WT A1-III mixed with Freund's incomplete adjuvant (FIA). Ten days after the first and fourth boost, serum was collected and antibody titers were evaluated by determining the serum dilution required to produce a 50% ELISA signal against the WT immunogen. Polyclonal A1-III specific IgG antibodies were purified from immune serum using an AminoLink Plus A1-III affinity column prepared from purified WT-his enzyme as per the manufacturer's instructions. The purified primary antibody was aliquoted and stored at 700 µg/ml, −20°C in PBS. ELISAs were performed in high binding 96-well plates using purified alginate lyase enzymes, polyclonal rabbit IgG antibody, secondary goat anti-rabbit HRP conjugate and ABTS for detection. Dose response curves were fit to the data to obtain EC_50_ values (half the maximal effective concentration of IgG). All ELISAs were performed in triplicate. The immunoreactivity of the PEGylated variants was defined as the ratio of the WT-his EC_50_ to the EC_50_ of the corresponding PEGylated enzyme. Equivalent binding of the WT-his and PEGylated variants to the 96-well ELISA plates was verified by activity assays of enzyme solutions pre- and post-binding. No statistically significant difference in the fraction of bound enzyme was observed for PEGylated or non-PEGylated enzymes (data not shown).

### Human scFv Antibody Binding Studies

The immunogenicity of WT-his and A53C-his-PEG was further assessed using an *in vitro* assay that scores the relative reactivity of a protein of interest towards a human antibody fragment library displayed on the surface of yeast^1^ [34]. Briefly, WT-his and A53C-his-PEG were biotinylated as per the manufacturer's instructions (Pierce Biotinylation Kit). Magnetic streptavidin beads (Invitrogen, Carlsbad, CA) were coated separately with each biotinylated enzyme overnight at 4°C. WT-his coated beads were combined with A53C-his-PEG coated beads, and the mixture was incubated with yeast expressing the human scFv library [25]. Following this binding step, the beads were magnetically separated and unbound yeast were removed by aspiration. The remaining bead:yeast mixture was placed in selective yeast growth media, and the selected yeast cells were regrown, induced, and selected against pooled beads a second time. This affinity-selected yeast population was regrown, induced and then independently incubated for one hour at 4°C with either WT-his or A53C-his-PEG coated beads. The beads were magnetically separated, and unbound yeast were aspirated and discarded. The yeast:bead mixtures were then resuspended in 1 ml of PBS, and a 50 µl aliquot was removed for serial dilution and plating on selective media to determine the number of yeast initially bound to each set of beads (“wash 1” population). The remainder of the resuspended yeast:bead mixture was then gently agitated at 4°C for 15 minutes, and the wash process was repeated twice more to generate “wash 2” and “wash 3” cell populations. The number of yeast binders to each protein target was quantified by plating serial dilutions of each wash population on selective growth media. Following a 2-day outgrowth, the number of cfu on each plate were determined and used to back calculate the total number of bead-bound yeast after each wash step. These values provide a relative metric for comparing the immunoreactivity of WT-his and A53C-his-PEG proteins towards a human scFv antibody fragment repertoire.

Following these initial studies, which yielded a relative count of antibody fragments capable of recognizing each individual enzyme, the cross-reactivity of yeast isolated against each protein target was evaluated. This analysis involved regrowth and induction of the wash 3 yeast populations, and subsequent magnetic bead selection against both protein targets in parallel.

A more detailed description of the methods for the human scFv antibody fragment studies is provided as Text S1.

### Biofilm Disruption Assays

The capacity of the alginate lyase enzymes to disrupt bacterial biofilms was assessed *in vitro*. Briefly, mucoid Xen5 *P. aeruginosa* (Caliper Life Sciences, Hopkinton, MA) cultures were grown for 19 hours in 3 ml of TSB media at 37°C. Bacteria were then subcultured at a 1∶5 ratio into fresh TSB media, and 100 µl aliquots were added in replicate to 96-well plates. Plates were covered with a gas permeable adhesive strip, and incubated without shaking for 20 hours at 37°C. Following biofilm growth, culture media and planktonic bacteria were shaken from the wells, and the remaining biofilms were rinsed with double distilled water. The adherent biofilms were treated with 200 µl aliquots of 1 mg/ml alginate lyase in 20 mM NaH_2_PO_4_ pH 6.5. Buffer only was used as a no treatment control. Each treatment was done in triplicate. Reactions proceeded at room temperature for 1 hour, after which enzyme solutions were shaken from the plate, and wells were again rinsed with double distilled water. Subsequently, 100 µl aliquots of 0.1 µg/ml ConA-HRP were added to all wells. Blank wells containing no biofilm were used as a background control. The ConA-HRP lectin was allowed to bind for 1 hour at room temperature, and the solution was then shaken from the plate followed by rinsing with double distilled water. Finally, 100 µl of ABTS substrate was added to all wells, and reactions were incubated for 15 minutes at room temperature before being quenched with 100 µl of 1% SDS. The absorbance of each well was measured at 405 nm, background signal from the blank wells was subtracted from experimental wells, and the percent decrease in biofilm was calculated by normalizing the signal from enzyme treated wells to that of wells receiving no enzyme treatment. Additional experiments directly monitored degraded alginate reaction products in treated biofilm supernatants, and the resulting data supported the conclusions drawn from the ConA-HRP lectin studies.

## Supporting Information

Movie S1 **Sites of cysteine substitution.** Ribbon diagram of alginate Lyase A1-III (PDB file 1HV6). A trisaccharide reaction product is bound in the active cleft and shown as a grey ball and stick model. Amino acid residues targeted for cysteine substitution are shown in space filling mode, and are color coded as follows: S32C = Red, A41C = Orange, A53C = Green, A270C = Yellow, and A328C = Purple.
(MPG)

Text S1 **Human scFv library binding experiments.**
(DOC)
