# Supplementary material for: Genetically Engineered Alginate Lyase-PEG Conjugates Exhibit Enhanced Catalytic Function and Reduced Immunoreactivity
Source: PLoS One. 2011 Feb 14;6(2):e17042. doi: 10.1371/journal.pone.0017042 (PMC3038863; doi:10.1371/journal.pone.0017042)
Supplement: Text S1 — Human scFv library binding experiments. (DOC) [file pone.0017042.s002.doc]

**Supplemental Text S1: Human scFv Library Binding Experiments**

John W. Lamppa^1^, Margaret E. Ackerman^1^, Jennifer I. Lai^2^, Thomas C. Scanlon^1^, Karl E. Griswold^1,3,4,*^

Thayer School of Engineering^1^, Dartmouth College, Hanover, NH; Department of Biological Engineering^2^, Massachusetts Institute of Technology, Boston, MA; Department of Biological Sciences^3^, Program in Molecular and Cellular Biology^4^, Dartmouth College, Hanover, NH

*Corresponding Author: Karl E. Griswold, PhD, Dartmouth College, 8000 Cummings Hall, Hanover, NH 03755. E-mail: [karl.e.griswold@dartmouth.edu](mailto:karl.e.griswold@dartmouth.edu)

**Protein Biotinylation**

WT-his and A53C-his-PEG were biotinylated with Sulfo-NHS-LC-Biotin essentially following the manufacturer’s recommendations (Pierce Scientific), but the molar excess of biotinylation reagent was adjusted to minimize excessive biotinylation (targeting 1-3 biotins per protein). Free biotin was removed by buffer exchange into phosphate buffered saline (PBS) using 10 kDa cutoff Amicon ultrafiltration spin columns (Millipore). Successful biotinylation was confirmed by western blot, and molecular biotinylation levels were assessed with a biotin quantification kit (Pierce).

**Magnetic Bead Coating**

Twenty-five microliter aliquots of biotin binder magnetic beads (~10^7^ beads, Invitrogen) were washed twice by placing the beads on a magnet for 5 minutes, aspirating the supernatant, and resuspending in 0.1% BSA in PBS. The washed beads were then resuspended in 50 µl of ~2.5 µM target protein, the mixture was diluted with 500 µl PBS, and the sample was mixed gently for ~12 hours at 4°C. Unbound protein was removed by washing the beads twice in PBS using magnetic separation as described above. Saturation of the bead surfaces had been confirmed by earlier experiments in which beads were coated with increasing concentrations of biotinylated protein followed by fluorescent staining with streptavidin-PE and quantitative analysis by flow cytometry as described previously [[1](#_ENREF_1)]. The resulting mean fluorescence for each bead preparation was plotted verses the labeling concentration of protein, and saturating protein concentrations were used in all subsequent bead coating experiments.

**Yeast Library Preparation**

The scFv library was grown and induced as described previously [[2](#_ENREF_2),[3](#_ENREF_3)]. The surface displayed scFvs were fused to a C-terminal c-myc tag, which facilitated flow cytometric analysis of the antibody expression levels. Prior to each experiment, an aliquot of the induced library population was stained with chicken anti-c-myc IgY and then Alexa Fluor 488 goat anti-chicken IgG (Invitrogen) to confirm scFv expression at the single cell level.

Prior to the immunogenicity assays, the yeast library was depleted of streptavidin and biotin binding scFvs. Briefly, 4x10^9^ yeast cells were induced and then incubated with 100 µl of magnetic streptavidin beads at 4°C for 1 hour. The beads were then magnetically separated, and the unbound yeast in the supernatant were used in subsequent experiments. A similar negative selection was performed against beads coated with heavily biotinylated BSA in order to deplete biotin-binding scFvs from the population.

**Immunogenicity Assays**

The negatively selected yeast were incubated for one hour at 4°C with pooled beads: one-half of which were saturated with WT-his and the other half saturated with A53C-his-PEG. The yeast:bead slurry then placed on a magnet for 5 minutes, and the supernatant containing unbound yeast was aspirated and discarded. The beads were removed from the magnet, resuspended in 1 ml of PBS, diluted into 50 ml of growth media, and grown and induced as described above. This process was repeated once more with the pooled beads. Note that scFv expression level is a critical variable in the bead-based selections, and expression levels can be sensitive to subtle differences in culture conditions. The initial selections were therefore performed with pooled beads, so as to ensure that the immunogenicity of the wild type and PEGylated proteins were ultimately compared using the same prescreened yeast population. Following outgrowth and induction from the second pooled bead selection, truncated scFvs were removed from the population by fluorescently probing for the *C*-terminal c-myc tag and sorting c-myc positive (i.e. full length scFvs) yeast on a FACSAria cell sorter. Note: prior to all outgrowth steps, the size of the selected yeast population was determined by plating serial dilutions and enumerating colony forming units (cfu). To ensure adequate coverage of the selected clones, a 10-fold oversampling was employed in subsequent experiments.

Yeast cells expressing full length scFvs from the pooled bead selections were grown and induced as described above. A 10-fold oversampling of the population was then incubated for 1 hour at 4°C with separate 25 µl aliquots of either WT-his coated beads or A53C-his-PEG coated beads. The beads were magnetically separated, and unbound yeast were aspirated and discarded. The yeast:bead mixture was then resuspended in 1 ml of PBS, and a 50 µl aliquot was removed for serial dilution and plating on selective media to determine the number of yeast initially bound to each set of beads (wash 1 population). The remainder of the resuspended yeast:bead mixture was then gently agitated at 4°C for 15 minutes, and the beads were subsequently magnetically separated as above. Unbound yeast were aspirated and discarded, bound yeast and beads were resuspended, and an aliquot was removed for plating (wash 2 population). This process was then repeated a third time, and the final bead-bound population was retained for cross-reactivity analysis. Plated bead dilutions were incubated for 2 days at 30°C, and cfu were counted. Two separate dilutions could typically be fully enumerated (between 5 and 200 colonies), and the respective counts were averaged to back calculate the number of bead-bound yeast after each wash step.

**Cross-reactivity Analysis**

The wash 3 yeast populations isolated against WT-his or A53C-his-PEG were grown, induced, and bead-selected against their original target as well as the other protein target separately. Binding counts were determined as described above.

**Data Analysis**

All plating and cfu enumeration was performed in triplicate. The average and standard deviation of 3 replicates is presented for each experiment. Statistical significance was determined with a two-tailed t-test.

1. Ackerman M, Levary D, Tobon G, Hackel B, Orcutt KD, et al. (2009) Highly avid magnetic bead capture: an efficient selection method for de novo protein engineering utilizing yeast surface display. Biotechnol Prog 25: 774-783.

2. Chao G, Lau WL, Hackel BJ, Sazinsky SL, Lippow SM, et al. (2006) Isolating and engineering human antibodies using yeast surface display. Nat Protoc 1: 755-768.

3. Feldhaus MJ, Siegel RW, Opresko LK, Coleman JR, Feldhaus JM, et al. (2003) Flow-cytometric isolation of human antibodies from a nonimmune Saccharomyces cerevisiae surface display library. Nat Biotechnol 21: 163-170.
